# Acute kidney injury due to direct infiltration by lymphoplasmacytic lymphoma secreting IgG paraproteins: A case report

**DOI:** 10.1097/MD.0000000000029449

**Published:** 2022-06-17

**Authors:** Seongmin Kim, Wooram Bae, Jungyoon Choi, Tae Won Lee, Dae Hyun Song, Eunjin Bae, Ha Nee Jang, Se-Ho Chang, Dong Jun Park

**Affiliations:** a Department of Internal Medicine, Gyeongsang National University Changwon Hospital, Changwon, South Korea; b Department of Pathology, Gyeongsang National University Changwon Hospital, Changwon, South Korea; c Department of Pathology, Gyeongsang National University College of Medicine; d Department of Internal Medicine, Gyeongsang National University College of Medicine; e Department of Internal Medicine, Gyeongsang National University Hospital, Jinju, South Korea; f Institute of Health Science, Gyeongsang National University, Jinju, South Korea.

**Keywords:** acute kidney injury, IgG, LPL, paraprotein, Waldenström's macroglbulinemia

## Abstract

**Introduction:**

Waldenström's macroglobulinemia is a lymphoplasmacytic lymphoma (LPL) associated with a monoclonal immunoglobulin M protein. Although acute kidney injury (AKI) due to immunoglobulin M paraprotein infiltration into the renal interstitium has been reported, there has been no report of AKI with invasion of the immunoglobulin G paraprotein into the renal interstitium in a patient with LPL.

**Patient concerns:**

A 65-year-old male was admitted to our hospital with fatigue and decreased renal function. He complained of a 3-kg weight loss in the last 3 months.

**Diagnosis:**

The initial blood urea nitrogen and serum creatinine levels were 55.9 and 1.83 mg/dL, respectively. Serum protein electrophoresis revealed a monoclonal component (3.5 g/dL) in the gamma region and immunofixation electrophoresis showed an immunoglobulin G kappa monoclonal protein. Renal pathology revealed that CD3–CD20+ CD138+ lymphoid cells had infiltrated the renal interstitium. A bone marrow biopsy was compatible with LPL.

**Interventions:**

Intravenous methylprednisolone (1 mg/kg) was administered after confirming the renal pathological findings.

**Outcomes:**

Serum creatinine decreased to 0.8 mg/dL 14 days after treatment

**Conclusions:**

Physicians should recognize LPL secreting various immunoglobulins as a possible cause of AKI when renal failure of unknown etiology and serum immunoglobulin paraprotein is present. A kidney biopsy should be performed for definitive diagnosis and appropriate management.

## 1 Introduction

Lymphoplasmacytic lymphoma (LPL) is a low-grade B-cell lymphoproliferative neoplasm characterized by small lymphocytes and monoclonal immunoglobulin M (IgM) monoclonal gammopathy. LPL is an extremely rare neoplasm, with an annual incidence of 3 to 4 cases per million people.^[1–4]^ The abnormal cells seen in patients with LPL have features of lymphocytes and plasma cells, and produce large amounts of abnormal antibodies called “paraproteins”.^[1,4]^ In most cases of LPL, IgM is the paraprotein. LPL with IgM detected on a blood test is called Waldenström's macroglobulinemia (WM).^[5]^ LPL rarely produces a paraprotein from different types of antibodies (usually immunoglobulin G [IgG]), but in such cases it usually produces IgM and IgG.^[6]^ Extranodal involvement, including of the gastrointestinal tract, lung, liver, spleen, skin, central nervous system, and kidney, is rare in LPL.^[1,3,4]^

The spectrum of renal diseases associated with LPL is continuously expanding with improvements in diagnostic technology. LPL-related nephropathies include characteristic intracapillary deposits of IgM with or without cryoglobulinemia, AL amyloidosis, and infiltration of the interstitium by neoplastic lymphoplasmacytic cells. Rare cases of immunotactoid and nonamyloid fibrillary glomerulopathy, cryoglobulinemia-related glomerulonephritis (GN), and crescentic GN have been reported.^[7–10]^ Although some cases of renal failure due to direct invasion of IgM-secreting monoclonal cells have been reported, there has been no report of acute kidney injury (AKI) due to direct infiltration by IgG-producing LPL. We provide the first report of a case of LPL accompanied by AKI with direct invasion of neoplastic cells secreting an IgG paraprotein.

## 2 Ethical statement and consent

Written informed consent was obtained from the patient for publication of their case report and any accompanying images. The study protocol was approved by the Institutional Review Board of Gyeongsang National University Changwon Hospital (IRB no. 2022-03-016).

## 3 Case report

A 65-year-old male with previous benign prostate hypertrophy was admitted to our hospital with fatigue and decreased renal function. He had been an office worker and retired 3 years ago. He had undergone medical check-ups over the last year and denied a history of diabetes mellitus or hypertension. His serum creatinine level was last measured at 0.8 mg/dL, according to medical reports. He complained of a 3-kg weight loss in the last 3 months. He did not complain of fever, oliguria, skin rash, or a change in urine color at admission. He had not taken non-steroidal anti-inflammatory drugs, toxins, or Chinese herbal medicines, but had been administered medicines for benign prostate hypertrophy.

His initial vital signs were blood pressure of 100/60 mm Hg, heart rate of 78 beats/minute, respiratory rate of 21 breaths/minute, and body temperature of 36.5 °C. His conjunctivae were mildly anemic and the sclerae were not icteric. Lymph nodes were not palpated on either side of the neck. No abnormal sounds were audible on chest auscultation, and the heartbeat was regular with no murmur. No organomegaly was present in the abdomen, and bowel sounds were audible. No pretibial pitting edema was observed on either lower extremity and no palpable lymph nodes were detected in either inguinal area. No skin color changes were evident on the body.

The blood urea nitrogen and serum creatinine levels were 55.9 mg/dL (normal range: 8.0–20.0 mg/dL) and 1.83 mg/dL (normal range: 0.51–0.95 mg/dL), respectively, at admission. The hematocrit and hemoglobin levels were 22% (normal range: 36–48%) and 7.0 g/dL (normal range: 12–16 g/dL), respectively. The platelet count was 180 × 10^9^/L (normal range: 130–400 × 10^9^/L). Other laboratory tests revealed a total protein of 9.5 g/dL (normal range: 6.6–8.7 g/dL), albumin of 3.5 g/dL (normal range: 3.5–5.2 g/dL), calcium of 8.1 mg/dL (normal range: 8.6–10.2 mg/dL), phosphorus of 3.1 mg/dL (normal range: 2.7–4.5 mg/dL), and lactic dehydrogenase of 151 U/L (normal range: 140–271 U/L). The C3 and C4 levels were 92 mg/dL (normal range: 90–180 mg/dL) and 32.9 mg/dL (normal range: 10–40 mg/dL), respectively. The IgG, immunoglobulin A, and IgM levels were 3825 mg/dL (normal range: 700–1600 mg/dL), 26.6 mg/dL (normal range: 70–400 mg/dL), and 29.9 mg/dL (normal range: 40–230 mg/dL), respectively. The markers for hepatitis B and C viruses were normal. Anti-nuclear antibody, anti-neutrophilic cytoplasmic antibody, and anti-glomerular basement membrane antibody were negative. Cryoglobulins were absent. A urinalysis (dipstick method) revealed − protein, 2+ blood, and–glucose. Microscopy revealed 10 to 29 red blood cells/high power field.

**Figure 1:**
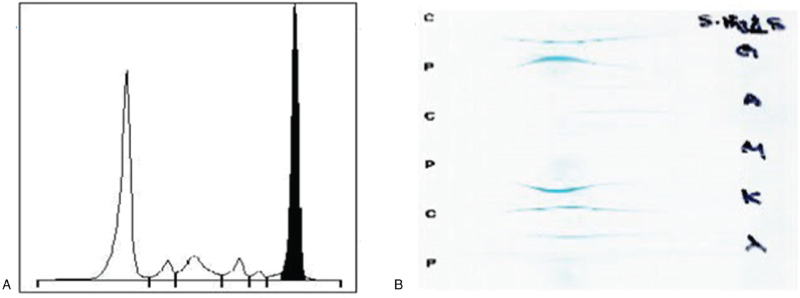
Serum protein electrophoresis and immunoelectrophoresis showing ‘M’ peak in gamma region (A) and IgG kappa monoclonal protein (B).

**Figure 2:**
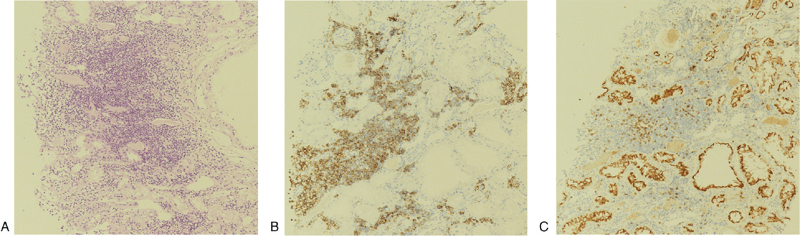
Histopathologic findings of renal biopsy. Light microscopy revealed moderate and diffuse lymphocytic infiltration into interstitium (x100, H&E) (A). Immunohistochemistry staining showing diffusely positive CD 20 (B) and focally CD 138 (C), respectively.

**Figure 3:**
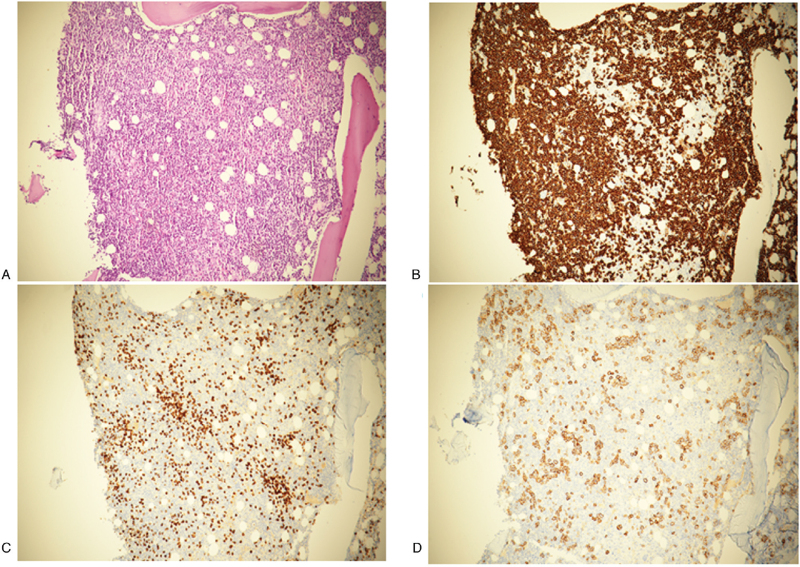
Histopathologic findings of bone marrow. Light microscopy showing increased atypical lymphoid cell aggregates (x100, H&E) (A). Immunohistochemistry staining for CD 20 (B), CD 138 (C), and CD 3 (D).

Serum protein electrophoresis (EP) detected a monoclonal component (3.5 g/dL) in the gamma region and immunofixation EP showed an IgG kappa monoclonal protein (Fig. 1). The serum-free kappa light chain level was 855.5 mg/L (normal range: 3.3–19.4 mg/L), the free lambda light chain level was 8.8 mg/L (normal range: 5.7–26.3 mg/L), the difference in the free light chain was 846.7, and the kappa/lambda ratio was 97.5. Ultrasound-guided renal biopsy was performed with suspicion of multiple myeloma (MM). Kidney biopsy showed that the glomeruli were normal and there was no positive immunoglobulin A, IgM, IgE, or IgG immunofluorescence, although CD3–CD20+ CD138+ lymphoid cells had infiltrated the renal interstitium (Fig. 2). Electron microscopy did not reveal any electron-dense deposits in the glomerular basement membrane and showed normal podocyte foot processes. Congo red staining was negative. These findings were not consistent with MM. A subsequent bone marrow biopsy was consistent with LPL. Atypical lymphoid cells increased (73.1%), and these lymphoid aggregates were usually composed of CD20- and CD138-positive cells (Fig. 3); CD5 and CD10 were rarely expressed. The *in situ* hybridization assay was positive for kappa, but not lambda (data not shown). The bone marrow karyotype was normal. No lymph node enlargement, splenomegaly, or hepatomegaly were detected on chest, abdominal, or pelvic computed tomography.

Intravenous methylprednisolone (1 mg/kg) was administered after confirming the renal pathology findings. Serum creatinine decreased to 0.8 mg/dL 14 days after treatment. Chemotherapy was recommended for the LPL just after completing the work-up, but the patient wanted a second opinion and was transferred to a tertiary hospital located in Seoul, South Korea. One year later, he revisited our outpatient clinic for an evaluation of renal function. He had undergone 6 months of chemotherapy and his condition had improved. His creatinine level was measured as 1.2 mg/dL in our hospital.

## 4 Discussion

We present a unique case of AKI due to direct infiltration by IgG-secreting LPL. Our patient was initially suspected to have MM. However, renal biopsy did not reveal typical findings of MM. The presence of an IgG monoclonal peak, but not IgM, on serum immuno-EP excluded WM. A bone marrow examination played a decisive role in confirming the diagnosis. This report is significant in showing that AKI can occur in a patient with IgG-secreting LPL, although this is extremely rare.

Renal manifestations are most likely related to abnormal monoclonal protein and/or tissue infiltration by underlying lymphoplasmacytic cells.^[8]^ The most common clinical renal manifestations in WM are mild proteinuria and microscopic hematuria. Bence-Jones proteinuria is rarely present compared to cases of MM.^[11]^ Some cases (<7%) present with nephrotic syndrome.^[10]^ Although the true incidence of WM-associated renal disease is unknown, one autopsy report revealed that 3.8% to 7.4% of WM patients have kidney disease.^[12]^ A recent large retrospective study that performed renal biopsies suggested a cumulative incidence of 5.1% at 15 years.^[10]^ A study conducted at another center showed kidney involvement in 8% of patients with WM.^[13]^ The rate of progression to end-stage renal disease is <3% in these patients.^[10]^

The wide variety of WM-related renal pathologies, such as AL-amyloidosis, non-amyloid GN, and tubulointerstitial diseases, caused by direct infiltration may be attributable to the different pathological processes associated with LPL tumor cells, IgM paraproteins, and the light chains, whereas minimal change in disease, focal segmental glomerulosclerosis, thrombotic microangiopathy, and membranous GN are associated with non-paraprotein mediated processes, probably indicating paraneoplastic syndrome.^[9–12]^ As in our case, direct infiltration of clonal B cells into the tubulointerstitium is an AKI mechanism. One study showed that severe tumor parenchymal infiltration in the absence of IgM deposits or light chain casts is the most common kidney pathological finding in patients who present with AKI.^[7]^ Higgins et al also reported tubulointerstitial disease in 14% of patients in their large Mayo Clinic series, where infiltration of lymphoma was the most common etiology.^[9]^ Uppal et al revealed that tubulointerstitial disease associated with WM was due to direct infiltration in 15.9% of patients with kidney disease.^[8]^ However, the association between LPL-secreting IgG and renal disease has not been established due to a paucity of data.

The most important disease in the differential diagnosis of WM is MM. Renal insufficiency occurs in up to 40% of patients with MM and is associated with adverse outcomes,^[14]^ whereas renal insufficiency occurs in only 5% of patients with WM.^[10]^ The MM pathology is cast nephropathy,^[14]^ whereas the kidney pathologies in WM are highly variable.^[10]^ These differences could be due to several distinct disease characteristics. Hypercalcemia is a known contributor to renal failure in MM, but none of our patients with WM-related nephropathy had hypercalcemia. The relationship between the burden of the free light chain and renal disease has not been established, because of a lack of literature data. The physicochemical properties of the various paraproteins, and the specific immunological activity of tumor clones, may influence the pattern of renal injury.^[10]^ In particular, IgM MM may be differentiated from WM by bone marrow biopsy, flow cytometry, the lack of organomegaly, and the presence of osteolytic lesions.

The symptoms and signs of WM range from vague symptoms, such as fatigue, weight loss, and anorexia, to more specific ones including anemia/thrombocytopenia, hyperviscosity, and hyperviscosity-related neurological, vascular, and hemorrhagic symptoms.^[1–4]^ The most common symptom is fatigue related to anemia, whereas the most common renal manifestations are mild proteinuria and microhematuria.^[11]^ Hepatomegaly occurs in 20%, splenomegaly in 15%, and lymphadenopathy in 15% of patients.^[2]^ The vague and atypical symptoms and signs do not make the diagnosis easy, particularly in the elderly population. The differential diagnosis of renal failure in these patients should include WM-unrelated renal diseases due to various underlying morbidities. Therefore, a kidney biopsy should be considered when there is an unexplained reduction in renal function, despite the absence of a simple urinalysis. Our patient initially complained of fatigue and underwent a routine check, including blood work and urinalysis. The existence of renal complications is a potential indicator of the need to initiate therapy.^[15]^ In addition, the diagnosis of renal disease by biopsy affects clinical management and treatment choices.^[16,17]^

Few data are available in the literature regarding the prognosis and outcomes of WM-related nephropathy.^[7–10]^ Chauvet et al reported a renal response in all 7 patients who achieved complete and/or very good hematological responses with therapy, but 6 of 35 patients progressed to end-stage renal disease. A renal response to chemotherapy occurred in all seven patients who achieved a complete and/or very good response.^[7]^ In a study by Vos et al, the median overall survival time of patients with biopsy-proven renal disease was shorter than that of the rest of the cohort, and patients with stable or improved kidney function after treatment had better survival.^[10]^ Higgins et al showed that patients with nonamyloid-related glomerulopathy have a longer median lifespan and higher kidney survival rate than those with amyloid-related glomerulopathy (160.5 vs. 64.4 months and 109 vs. 27 months, respectively). Kidney manifestations improved or stabilized in 47% of the patients (most commonly with nonamyloid glomerulopathy and least commonly with tubulointerstitial nephropathies).^[9]^

## 5 Conclusions

Physicians should recognize LPL secreting various IgGs as a possible cause of AKI in cases of renal failure of unknown etiology and the presence of serum IgG paraprotein, and perform kidney biopsy for definitive diagnosis and appropriate management.

## Author contributions

**Conceptualization:** Seongmin Kim, Dong Jun Park.

**Data curation:** Ha Nee Jang, Dae Hyun Song.

**Formal analysis:** Tae Won Lee, Eunjin Bae.

**Investigation:** Ha Nee Jang, Wooram Bae.

**Methodology:** Jungyoon Choi, Wooram Bae.

**Supervision:** Se-Ho Chang, Dong Jun Park.

**Validation:** Tae Won Lee, Eunjin Bae, Dae Hyun Song.

**Writing – original draft:** Seongmin Kim.

**Writing – review & editing:** Se-Ho Chang, Dong Jun Park.
